# 3‐O Sulfated Heparan Sulfate as an Emerging Drug Target and Biomarker for Alzheimer’s Disease

**DOI:** 10.1002/alz.088959

**Published:** 2025-01-03

**Authors:** Chunyu Wang, Jian Liu, Lianchun Wang, Dylan Mah, Fuming Zhang

**Affiliations:** ^1^ Rensselaer Polytechnic Institute, Troy, NY USA; ^2^ University of North Carolina at Chapel Hill, Chapel Hill, NC USA; ^3^ University of South Florida, Tampa, FL USA

## Abstract

**Background:**

Heparan sulfate (HS) interacts with many important proteins. These interactions are primarily driven by electrostatics, with specificity determined by sulfation patterns. Although 3‐O‐sulfation is a rare modification in HS, several genome‐wide association studies (GWAS) revealed that the Hs3st1 gene, encoding HS‐3‐O‐sulfotransferase‐1, is significantly linked to late onset AD risk.

**Method:**

NMR, SPR, glycan array were used to study HS‐protein interactions. A novel LC‐MS/MS method was developed to quantify the low abundance 3‐O‐sulfated HS from human brain, with ^13^C labeled calibrants. Alexa‐488 labeled Tau and ApoE was used to assess tau cell surface binding and uptake in HS3ST‐1 knockout cellular lines.

**Result:**

We have demonstrated that tau, the major component of neurofibrillary tangles, specifically recognizes the 3‐O‐sulfation (3‐OS). In addition, ApoE, whose isoform ApoE4 is the most significant genetic risk factor for late onset AD, also interacts with 3‐OS. In addition, the affinity between ApoE isoform and heparin correlates with AD risk, with ApoE4 having the highest affinity to heparin, while the protective ApoE2 and Christchurch having significantly lower affinity. With a novel LC‐MS/MS method for quantifying 3‐O‐sulfation, our recent studies have further demonstrated a marked increase in 3‐O‐sulfated HS in AD brains.

**Conclusion:**

Taken together, our findings underscore the importance of 3‐OS in AD pathogenesis, suggesting its potential as a novel drug target and biomarker in Alzheimer’s disease.

